# Trends in river herring environmental DNA in two North Carolina river systems

**DOI:** 10.1371/journal.pone.0347206

**Published:** 2026-05-04

**Authors:** Seth M. Gibbons, Chase G. Spicer, Sara Roozbehi, Chequita N. Brooks, Cory S. Joyner, Michael S. Brewer, Roger A. Rulifson, Erin K. Field

**Affiliations:** 1 Department of Biology, East Carolina University, Greenville, North Carolina, United States of America; 2 North Carolina Aquarium on Roanoke Island, Manteo, North Carolina, United States of America; 3 North Carolina Wildlife Resources Commission, Raleigh, North Carolina, United States of America; 4 Louisiana Universities Marine Consortium, Chauvin, Louisiana, United States of America; University of Iceland, ICELAND

## Abstract

River herring (Blueback Herring [*Alosa aestivalis*] and Alewife [*A. pseudoharengus*]), were once abundant in North Carolina waters and were an economically important commercial fishery. Both populations have declined in recent history due to habitat loss, overfishing, and river alterations. Eastern North Carolina’s turbid and large river systems make traditional sampling difficult; thus, a rapid and accurate method for quantifying spawning populations is needed. The use of environmental DNA (eDNA) to detect river herring has been successfully applied to other watersheds across the Atlantic Coast. To determine if eDNA techniques could be applied to turbid watersheds to monitor river herring movement during the spawning season, weekly water sampling was performed at eight sites along the Neuse and Tar-Pamlico Rivers in North Carolina. Sampling for eDNA at two locations along the Neuse River were conducted in tandem with traditional electrofishing surveys to compare trends in river herring abundance between methods. The results indicate that both river herring eDNA concentrations and fish counts using electrofishing increased in the first three weeks of the spawning season. During the second half of the spawning season, eDNA concentrations remained high while fish abundances decreased. These results suggest that eDNA may be more consistent with electrofishing early in the spawning season while it may detect eDNA from juveniles, larvae, and persistent ambient eDNA later in the spawning season after adults have left the system. Sampling multiple locations along the rivers also indicated that the eDNA trends were consistent between different river systems. Sampling location where eDNA was collected mattered, as sites further upriver were more consistent in eDNA trends between river systems, and are likely better for long-term monitoring, compared to downstream locations. The results of this work help lay the foundation for the application of eDNA for future monitoring efforts in turbid waters, including North Carolina watersheds.

## Introduction

The analysis of environmental DNA (eDNA), the genetic material shed by an organism into its environment, is becoming a widespread tool in the field of fisheries biology, especially for determining the presence or absence of cryptic or imperiled fish species [1–6]. eDNA can be released into the environment through the sloughing of cells, production of waste material, release of gametes during the spawning season, and through washing of genetic material from the terrestrial environment into water [7]. Therefore, if the DNA of a certain organism is found in an environmental sample, then it can be inferred that this is a positive indication that the organism is, or was recently, present in the sampled system [8]. Studies, such as those of the Bigheaded Asian Carp (*Hypophthalmichthys* spp.), have shown that eDNA is influenced by environmental conditions and where sampling occurs [9]. Here, they found that eDNA was more concentrated in sediment than water. While this is better for overall detection of their presence, resulting eDNA concentrations in sediments are not thought to be reflective of populations at the immediate time of sampling as DNA can remain days to weeks after the organism itself has left the area suggesting it’s not an appropriate marker for real-time stock assessments due to the buildup and persistence of DNA over time. Therefore, the use of water samples to collect eDNA may be more representative of the recent presence of organisms in the area surrounding water collection sites. This technology has proved to be useful for monitoring when used in conjunction with other, traditional, sampling techniques for monitoring the current and projected future populations of aquatic organisms [6,10].

The term “river herring” applies to two species: Alewife (*Alosa pseudoharengus*) and Blueback Herring (*A. aestivalis*). The two anadromous species are grouped and managed together due to their similarities, both genetically and phenologically [11–12]. Historically, these two species were very common in all Eastern North Carolina (NC) watersheds; however, populations of both species have greatly declined [13]. In recent years, multiple applications have been submitted to the National Oceanic and Atmospheric Administration to have both species declared endangered. As of this writing, the listing as endangered species has not occurred. Some recovery actions that NC has enacted, including strict size and catch limits and a moratorium, seem to be positively affecting both populations, although the stock of river herring is currently still defined as depleted [14–15].

River herring in NC have been impacted by anthropogenic activities including habitat degradation, overfishing, and the construction of dams and other structures that block the passage of spawning fish [16]. There is also evidence to suggest that global climate change may have a negative impact on these populations [17–18]. The decreasing population sizes have also been observed to have a negative effect on genetic variability of river herring stocks [19]. Considering the multitude of issues hindering recovery of both species’ populations, it is crucial to identify efficient alternatives to electrofishing, which is currently the primary method of monitoring river herring populations. When sampling is done systematically across multiple years, electrofishing sampling can provide valuable data on the size and timing of a species’ spawning run; however, there are a number of factors that make this method more difficult in large, turbid river systems like those in Eastern North Carolina [6].

The river systems in Eastern NC have several characteristics that make traditional methods of counting spawning anadromous fish intractable. Visual counting of river herring in NC is difficult due to the highly turbid waters, which have extremely low visibility. Therefore, fish cannot be seen if they are not right at the surface. Electrofishing and sonar systems can be used to get fish counts, but the large geographic range of Eastern NC rivers and innumerable, unnavigable tributaries mean that these methods are inefficient and not feasible for effectively monitoring river herring spawning populations on an annual basis. Mass deployment of electrofishing boats with trained personnel or the mass installation of sonar-based systems would be needed in order to measure river herring populations across the Eastern part of the state during their spawning season, while the collection of water samples for eDNA can be done quicker and with fewer personnel in the field as all the fieldwork required by this method is the water collection. Further, the higher presence of organics in these waters can also impact eDNA persistence and concentrations much like sediment, indicating it’s important to assess eDNA techniques applications in these types of environments. Therefore, the development of a non-invasive, rapid, quantitative method using eDNA is a priority. Eastern NC watersheds represent a model system for studying these types of turbid, challenging environments which are common globally. Eastern North Carolina rivers are turbid, mostly due to the fact that they typically contain substrates made up of mostly sand and silt and historically large inputs from storm runoff and agricultural waste [20]. This fact combined with the fact that these systems are also mostly low-gradient streams with a slower flow mean that fine particles tend to remain longer in the system, rather than being swept away quickly [21].

River herring enter Eastern NC rivers to spawn during spring. Otherwise, adults live in the ocean, and only larval or juvenile fish could ostensibly contribute to the watershed eDNA profile [22–23]. For this reason, sampling during spring provides the best information on migration timing and spawning population size. This means the sampling window is as short as a few weeks. Currently, management agencies rely on weekly electrofishing surveys for river herring stock assessments in NC watersheds. Electrofishing surveys are common throughout this region; however, they cannot hope to cover the enormity of the tributary systems within this restrictive timeframe. Meanwhile, eDNA has been shown to be a reliable method for the detection of other fish species and it could prove to be a rapid and cost-effective option for management agencies to monitor river herring spawning populations in North Carolina, whether by itself or in tandem with traditional sampling techniques [24]. A study in Japan focused on another anadromous fish species, the Shishamo Smelt (*Spirinchus lanceolatus*), and found that trends in eDNA concentrations were indicative of the number of migrating fish [25]. These findings could be important to understanding river herring populations and their movements. A well-developed and field-tested methodology for detecting river herring eDNA has been used in other watersheds along the East Coast, with primary efforts in the Chesapeake Bay region [26–28], but it is important to understand how this method could be applied to North Carolina’s higher turbidity watersheds. If eDNA methodology is to be used for monitoring river herring populations in NC long-term, we will need to better understand where eDNA concentrates, how it disperses in the environment, and if it mimics the same trends in fish abundances measured by traditional electrofishing surveys.

Here, we evaluated the eDNA trends across two NC rivers (Neuse and Tar-Pamlico Rivers) as a first step in implementing eDNA as a monitoring tool for river herring spawning populations in NC. Further, as a turbid watershed it also sets the foundation for the application of eDNA techniques for anadromous fish in similar river systems globally. The analysis of eDNA abundance in large river systems has not been widely performed at this time, so more information is needed about what general trends may exist in detection up- and downstream [29]. This will aid in identifying locations best suited for sampling, how frequent sampling must occur, and what environmental factors will affect the fate of eDNA in these watersheds. In order to address our questions about monitoring methodology, we conducted weekly sampling at six locations across the two river systems throughout the spawning season to evaluate how eDNA concentrations change as the fish move through the rivers. By sampling two eastern NC river systems, we aim to determine how consistent these eDNA trends are between watersheds, providing a metric of feasibility in using eDNA methodology as a part of widespread national management efforts.

## Materials and methods

### Tandem River Herring Sampling with Traditional Electrofishing Surveys and Alternative eDNA Tools in the Neuse River

**Fig 1:**
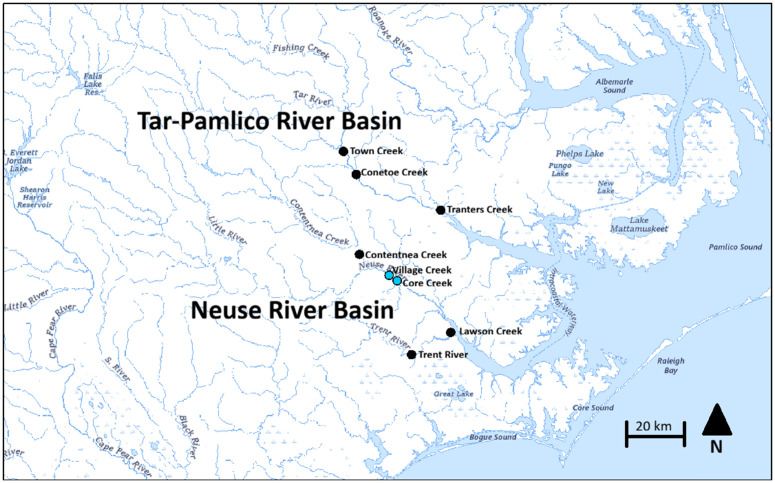
Sampling locations for river herring eDNA analyses in both the A) Tar-Pamlico River, NC and B) Neuse River, NC indicated by black markers. Sites where both electrofishing and eDNA sampling were performed in concert are indicated by blue markers. Upper right insert shows the location of these watersheds in relation to the state of North Carolina. Image from the U**.**S. Geological Survey’s Geospatial Program, The National Map [30] modified with MS Paint tool (Windows 11).

Two sites on tributaries of the Neuse River, Core Creek (35.2898818 N, −77.2849657 W) 97.36 km from the mouth of the river and Village Creek (35.3078153 N, −77.3017298 W) 99.14 km from the river mouth (Fig 1), were sampled over seven weeks in 2018 (February 26-April 12) and four weeks in 2019 (March 19-April 11). The electrofishing and eDNA sampling efforts were performed in tandem in order to provide information on whether or not there is a correlation between fish count and eDNA concentrations. This will aid in our understanding of the utility and potential application of a new monitoring method (i.e., eDNA) compared to traditional stock assessments (i.e., electrofishing) in turbid waters, such as those in eastern North Carolina. Specifically, weekly water samples were collected just prior to the start of electrofishing providing a lag time on the order of minutes. They were collected prior to electrofishing in order to minimize the risk of cross-contamination during electrofishing. These two sites were sampled with support from the North Carolina Wildlife Resources Commission (NCWRC) using a boat-mounted electrofishing unit (Smith-Root 7.5 GPP) set to 1,000 volts, 4–6 amps, 120 Hz pulsed DC for approximately 900 sec along two transects with one dip netter to collect both adult blueback and alewife herring [15]. This was conducted as part of the NCWRC annual river herring stock assessment surveys, carried out at the locations and dates where these surveys were occurring, and followed the agencies’ standard protocol. Replicate eDNA samples were collected alongside traditional electrofishing surveys weekly (see details below). eDNA sample collection was performed at the sites of the surveys at the same time as the electrofishing efforts. The number of river herring caught at each site was recorded and then compared with eDNA concentrations.

### Expanded regional eDNA Sampling of the Neuse and Tar-Pamlico river systems

Sampling efforts to collect river herring eDNA were expanded in 2019 in an effort to compare trends in eDNA concentrations between two NC river systems: the Neuse and Tar-Pamlico Rivers and at locations along each river system. This provides a better assessment of the eDNA trends across the eastern North Carolina region, expanding the sampling size of this study, and ensuring the trends observed are not limited to just the Neuse River system. These two rivers feed into the Pamlico Sound, and their basins cover a large part of Eastern NC with a combined basin area of 30,520 km^2^. Both the Neuse and Tar-Pamlico rivers are known spawning watersheds for river herring. Six tributaries of each system were sampled: three along the Neuse River and three along the Tar-Pamlico River (Fig 1). For the purpose of measuring distance to the river mouth, we considered the mouth of the Neuse River to be at Maw Point (35.15063 N, −76.53704 W) and the mouth of the Tar-Pamlico River to be at Roos Point (35.37285 N, −76.46998 W). The three Neuse River sites, from upstream to downstream in nautical kilometers, were Contentnea Creek (35.368578 N, −77.434325 W) 117 km, Trent River (35.009172 N, −77.218887 W) 86.9 km, and Lawson Creek (35.1017149 N, −77.0524829 W) 62.12 km. The Tar-Pamlico River sites sampled, from upstream to downstream in nautical kilometers, were Town Creek (35.790674 N, −77.548742 W) 123.28 km, Conetoe Creek (35.695555 N, −77.490519 W) 109.76 km, and Tranter’s Creek (35.5626824 N, −77.0864289 W) 62.44 km. The 2019 weekly sampling at the remaining sites took place over five weeks (March 24-April 17, 2019). Water collection at these sites was performed from docks or piers, both for ease of access and to ensure that sampling would be feasible for a single person. Sites along these streams with docks or piers were also chosen as opposed to sites without those structures, as any structure that allows for collection farther out into the stream is more representative of the stream’s flow and depth than a simple collection from a stream’s bank. Sampling from the center of the stream would be the preferred method; however, this was beyond the means of researchers at the time of sampling. See below for collection conditions and description.

This work was conducted under the approved ECU IACUC AUP #D353 and was conducted following all prevailing local, national, and international regulations and conventions, and normal scientific ethical practices. During this study, no fish were injured and all river herring were released after electrofishing.

### eDNA Sample Collection and Processing

At each site replicate 1-L water samples were collected below the water surface in sterile 2-L Nalgene bottles. Prior to sampling, bottles were sequentially washed three times with Alconox soap solution, three washes with a 10% bleach solution, rinsed three times with deionized water and allowed to dry for up to one week before the next sampling event. To control contamination during sampling, new nitrile gloves were worn at each site to prevent the transfer of eDNA between locations. Bottles were also rinsed three times with ambient water at each location prior to collecting a biological sample. Bottles were placed in a cooler with ice packs for immediate transport back to the lab to undergo filtering. For samples collected in tandem with electrofishing, these same protocols were used as well as some additional constraints. Specifically, bottles were kept in the closed cooler until arriving at the sampling site and immediately put back in after collection and were collected prior to electrofishing. Samples were then also transported back to the lab at ECU on ice in a cooler for filtering within two hours and immediately filtered.

The water samples were filtered through 0.45-µm cellulose nitrate filters housed in single use, Nalgene® Rapid-Flow™ Sterilization Filter Units (Thermo Fisher Scientific, Waltham, MA, USA). These single use filter units were used to minimize the risk of cross-contamination between samples filtered. The volume of water filtered varied from 500 mL to 1 L due to varying turbidity at each site and particles; sample volume was recorded so that results could be normalized to volume filtered. Filtering time was estimated to be less than one hour per sample. Filters were aseptically removed and stored at −80°C for downstream analyses.

The eDNA was extracted from the filter using the PowerSoil DNA extraction kit (Qiagen, Venlo, Netherlands). This kit was used as it provided higher yields (3x higher ng/uL per 1L water filtered) when directly compared to the commonly used Omega Biotek E.Z.N.A.® DNA/RNA Isolation Kit when replicate water samples were processed using both kits. This is likely due to the more stringent lysis and washing steps necessary to extract DNA from the turbid, organic-rich water that would affect downstream qPCR analyses. Water filtering, DNA extractions, qPCR setup, and qPCR analyses were all conducted in separate rooms to minimize the risk of cross-contamination between steps.

### Quantitative PCR methods

Quantitative PCR (qPCR) was performed to determine eDNA concentrations over time. These primers target part of a mitochondrial gene, cytochrome c oxidase subunit 1 (COX1) which is specific to both Alewife and Blueback Herring, equally amplifying DNA from both species [26]. The samples were subjected to sequential runs of qPCR in 96-well plates with 96 reactions. Each 20 µL reaction included 10 µl Bio-Rad SYBR Green Master Mix (Bio-Rad, Hercules, CA, USA), 1 µl each of 10 µM river herring-specific primers CO1_RH-F (5’- ATG AGC TTC TGA CTA CTT −3’) and CO1_RH-R (5′- GAT AGT TAG ATC GAC GGA −3′), 2 µl extracted DNA, and 6 µl of nuclease free water [26]. PCR thermal cycler conditions were 1 cycle of 45 seconds at 95°C, followed by 39 cycles of 5 seconds at 95°C, 15 seconds at 58°C and 10 seconds at 72°C. There were three technical replicates of each sample, standards, and negative controls in every run. Quantitative PCR (qPCR) standards were developed using fin-clips of blueback herring with concentrations spanning several orders of magnitude to ensure accurate quantification. Specifically, standards ranged from 2.13 × 10 ⁻ ¹ to 2.13 × 10 ⁻ ⁸ ng/µL and positives were confirmed within this range of all technical replicates. Results were normalized to ng river herring eDNA per 1 L river water filtered for each sample.

### Statistical analyses

A Pearson correlation analysis was utilized to examine relationships in eDNA concentration between sites utilizing MATLAB R2023 to quantify how well site trends correlated. In this analysis we compared eDNA concentration between five tributaries of the Neuse River from upstream (Town Creek, Contentnea Creek, Conetoe Creek, Trent River, Lawson Creek), and one tributary of the Tar-Pamlico River (Tranters Creek) that were sampled for a period of 5 weeks in 2018.

To determine the detection dynamics between eDNA concentration and fish counts, areas under curves (AUCs) were calculated for each site by year. Individual curves were generated for each site, year, and method (i.e., eDNA versus fish count) combination over time. Fish count and eDNA data were standardized by dividing the sample value by the sum of the samples included in the plot. AUCs were calculated for the entire plot and again for the region post the midpoint in sampling dates using the “auc” command from the R package (v. 4.3.3 macOS) gcplyr v.1.12.0 [31]. The persistence in detection between eDNA concentration and fish counts were calculated as the second half AUC divided by the total AUC for each plot (i.e., the proportion of AUC contained in the second half of the sampling dates).

To investigate the effect of upstream/downstream position on eDNA concentrations, non-linear mixed effects models (lme) were performed using the the “lme” command from the R package (v. 4.3.3 macOS) nlme v.3.1.164 [32]. Two models were employed: 1) eDNA concentration as a function of categorical position along streams (i.e., upstream, midstream, and downstream) with a random effect of river (Tar or Neuse), and 2) eDNA concentration as a function of distance to stream mouth in km with a random effect of river. Additional models were calculated with eDNA concentration as a function of the interaction between stream position (categorical or distance to mouth) and date with a random effect of river. Model selection was performed using the Bayesian Information Criterion (BIC) value tests. As a further test of the relationships in our data, permutation tests were performed for the categorical and distance to mouth approaches. Preferred models were used to analyze 1,000 randomizations of sample positions. The actual dataset’s AIC value was compared against the distribution of randomized models’ AIC values.

## Results and discussion

### Comparison of eDNA Concentration and Fish Counts Collected in Tandem in the Neuse River

**Fig 2:**
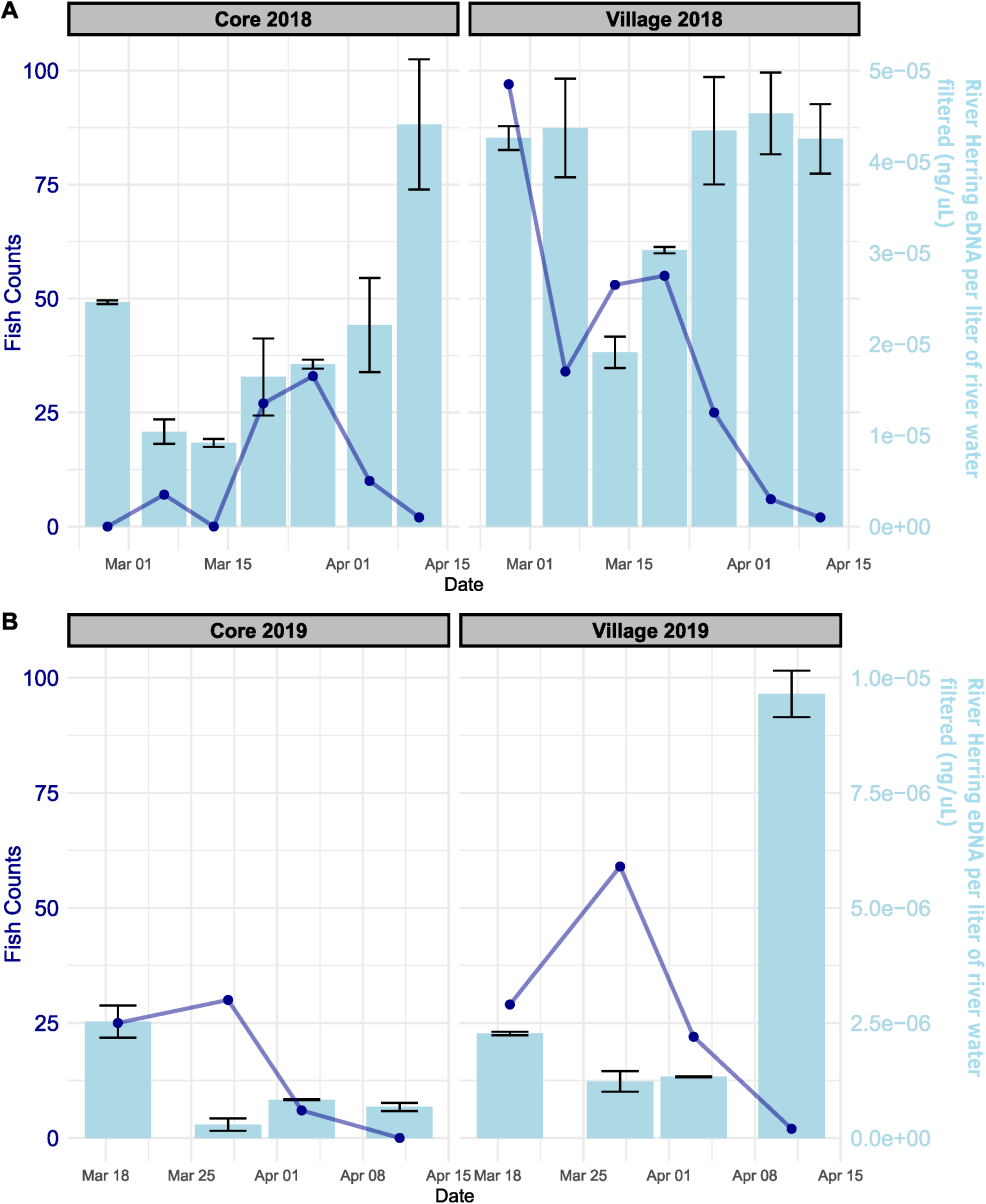
Individual spawning adult river herring fish abundances (dark blue, line with markers) and eDNA concentrations (light blue, column chart) across the 2018 and 2019 spawning seasons at the Core Creek and Village Creek sites along the Neuse River. eDNA concentrations have been normalized to ng river herring eDNA per 1L water filtered and standard errors for duplicate biological samples per sampling effort are included.

Results from Core and Village Creek sampling efforts indicated that while eDNA concentrations and fish counts increased during the first half of the spawning season, there was a desynchronization with very high eDNA concentrations compared to low fish counts later in the season (Fig 2, S1 Table, S1 Fig). Sampling efforts in 2019 confirmed this trend late in the season as eDNA concentrations remained high, even after adult river herring were no longer detected via electrofishing surveys (Fig 2B, S1 Table, S1 Fig). River herring eDNA concentrations reached higher peaks in 2018 than in 2019, but this is likely due to sampling only the second half of the spawning season in 2019 when the bulk of adult fish had likely already vacated the immediate area. The 2018 sampling period saw a slight decrease in eDNA concentrations on the March 13^th^ sampling date with concentrations remaining higher afterwards for the rest of the sampling season in both Core Creek and Village Creek. The 2018 peaks for both Core and Village Creeks occurred in April with Core Creek peaking on April 11^th^ and Village Creek peaking on April 4^th^. The actual river herring counts varied as well, with the eDNA peaks for the two systems coming on different weeks in 2018, but in the same week for 2019 (March 28^th^). The number of river herring collected in 2019 was lower overall than in 2018 in both systems.

**Fig 3:**
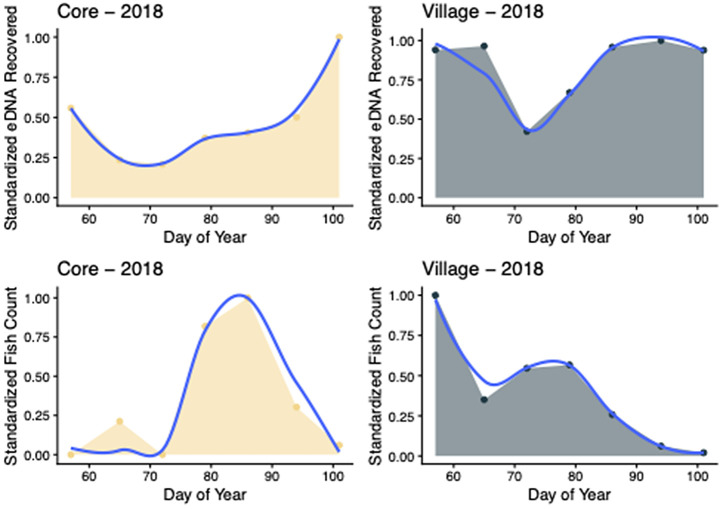
Area under curves (AUCs) calculated for spawning adult river herring fish abundances and eDNA concentrations across the 2018 spawning seasons at the Core Creek (yellow AUCs) and Village Creek (green AUCs) sites along the Neuse River. Fish count and eDNA data were standardized by dividing the sample value by the sum of the samples included in the plot.

AUC analyses supported the general finding that eDNA methods have overall higher sensitivity and greater detection potential later in the season. In Core 2018 samples, eDNA total AUC was 1.04 relative units higher than Fish Count total AUC, indicating similar levels of sensitivity across the season (Fig 3). Core 2018 eDNA found in the second half of sampling comprised 62.88% of the total AUC and was trending upward. Fish Count approaches yielded 73.58% in the later half of sampling but had returned to near zero detection at the end of sampling. Village 2018 eDNA total AUC was 19.71 relative units higher than the Fish Count data. The second half of eDNA sampling comprised 55.23% of the total, while Fish Counts in the second half made up only 25.89% of the total. Fish Counts reached near zero by the end of the sampling period, but eDNA detection persisted.

**Fig 4:**
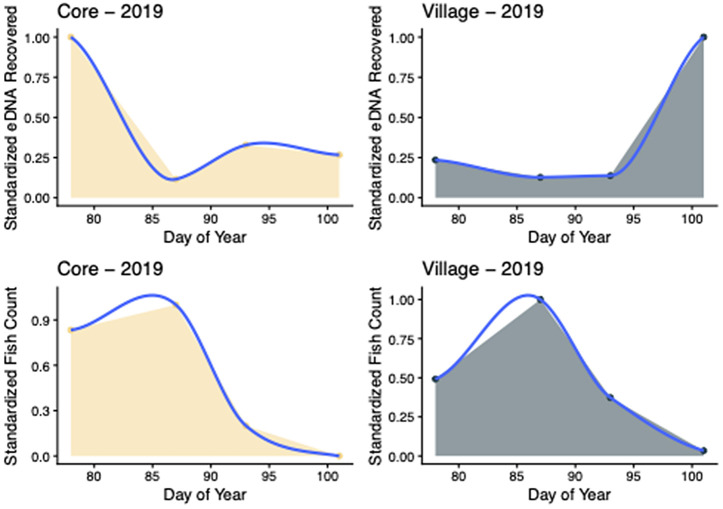
Area under curves (AUCs) calculated for spawning adult river herring fish abundances and eDNA concentrations across the 2019 spawning seasons at the Core Creek (yellow AUCs) and Village Creek (green AUCs) sites along the Neuse River. Fish count and eDNA data were standardized by dividing the sample value by the sum of the samples included in the plot.

Core 2019 eDNA AUC was 3.92 relative units lower than that of Fish Count methods, a result dissimilar to other site/year results (Fig 4). However, eDNA AUC in the second half was 37.34% of the eDNA total while Fish Count later half AUC was only 17.03% of the Fish Count total. Again, Fish Counts had reached near zero at the end of sampling while eDNA still detected fish presence. Similar to the 2018 results, Village 2019 eDNA AUC was 5.48 relative units lower than Fish Count methods. Similar to all other site/year results, eDNA detection was stronger in the second half of sampling with 71.50% of the AUC in the later half. In comparison, Fish Count later half AUC comprised only 27.22% of the total. These AUC results indicate generally similar levels of sensitivity across the full sampling period. However, eDNA approaches perform better later in the sampling period, allowing for detection of fish after the individuals have left the area.

All sampling efforts at Core Creek and Village Creek, with the exception of Core Creek in 2019, showed increased concentration of eDNA toward the end of the sampling sessions, even when the fish were no longer detected in the area based on electrofishing. All four sampling sites had an early and late season peak in both eDNA and electrofishing counts. One possible explanation that could explain the early season peak is that there could be multiple upstream movements of river herring within a single spawning season [33]. Other fish species have been known to migrate towards their spawning areas well before the start of spawning season; river herring could be practicing a similar strategy [34]. River herring do not necessarily spawn immediately upon reaching spawning sites, possibly delaying spawning until certain environmental conditions are right. The areas in which the fish spawn are typically more lentic habitats upstream. Although this study did not focus on specific spawning locations, eDNA gathered from these sampling efforts is still valuable as it is reflective of both migrating river herring and potential eDNA transport from migration and spawning activity further upstream. This means that future studies have the potential to help managers better understand exact spawning sites, as a higher eDNA concentration in an upstream site could indicate a closer activity to a spawning location. The late-season peak could be indicative of the fish farther upstream spawning, leading genetic material from these activities to be transported downstream to the sampling sites, even though the adult fish are no longer present. This hypothesis is supported by previous studies, which have shown that eDNA can be washed downriver nearly 10 km [35].

Elevated eDNA concentrations, despite low adult river herring counts from the traditional electrofishing surveys, suggest that there are other contributions to the eDNA signal besides adult fish actively moving through the area. The contribution of larval fish and gametes in the water on river herring eDNA concentrations is not known at this time and may be contributing to these high eDNA concentrations. A study done on another species of migratory fish indicated that the eDNA concentrations produced by the individuals of younger life stages of fish (larval and juvenile individuals) are lower than those produced by spawning adults, but these concentrations could still be significant if there are large numbers of these young fish present [36]. Young river herring have been known to exhibit patterns of migration where they re-enter rivers when they are still immature [37]. Other unknown environmental factors, including possibly less water flow during the later part of the season, could also be influencing the amount of detectable DNA in the system through disposal or degradation. These findings have implications for the future interpretation of eDNA results in these systems. Early season sampling appears to correspond more closely with the abundance of adult river herring, while sampling later in the season may reflect a combination of different life stages of the river herring, persistent eDNA, and the transport of eDNA from farther upstream in the system as opposed to simply the presence of adult herring. For these reasons, eDNA based monitoring seeking to assess river herring spawning runs should prioritize collections early in the season, and interpret any collections made later on in the season with more caution. In the Chowan River (S2 Fig) we have observed that river herring eDNA concentrations can reduce to below detection levels during the early summer months, indicating that it does not persist indefinitely throughout the year. Since there does appear to be some variation in spawning migration timing between years, it may be required to design eDNA monitoring efforts to cover a broader period of time, in order to help better contextualize eDNA sampling results. Since this variation occurs, the exact timing of river herring spawning runs is difficult to precisely predict. Study design changes could include environmental cues known to signal river herring movement (including water temperature) to assist in determining when to start sampling efforts. As far as late-season detection goes, future studies to monitor river herring movement in these systems may need to extend sampling time until it is clear that the adult fish have left the system.

The results of the weekly sampling conducted at Core and Village Creeks showed that yearly variation can occur in river herring eDNA concentrations. The differences between years could be caused by differences in lunar cycle, water temperature and water flow due to varying weather patterns and storms, which could affect when the herring enter the river systems to spawn since there is some evidence to support that warmer temperatures are a trigger for this behavior [38]. The spawning seasons of both species overlap (with Alewife moving into the rivers slightly earlier and Blueback Herring leaving the systems slightly later), but may be beginning to diverge due to warming waters in some systems [39]. It will be important to understand if these shifts are also taking place in Eastern North Carolina rivers. Between this variation and the imperiled status of the two species it is important that population monitoring occurs across the spawning season each year to get an accurate estimation of North Carolina river herring stocks.

### Expanded Sampling Efforts to Assess Regional eDNA Trends in the Neuse and Tar-Pamlico river watersheds

**Fig 5:**
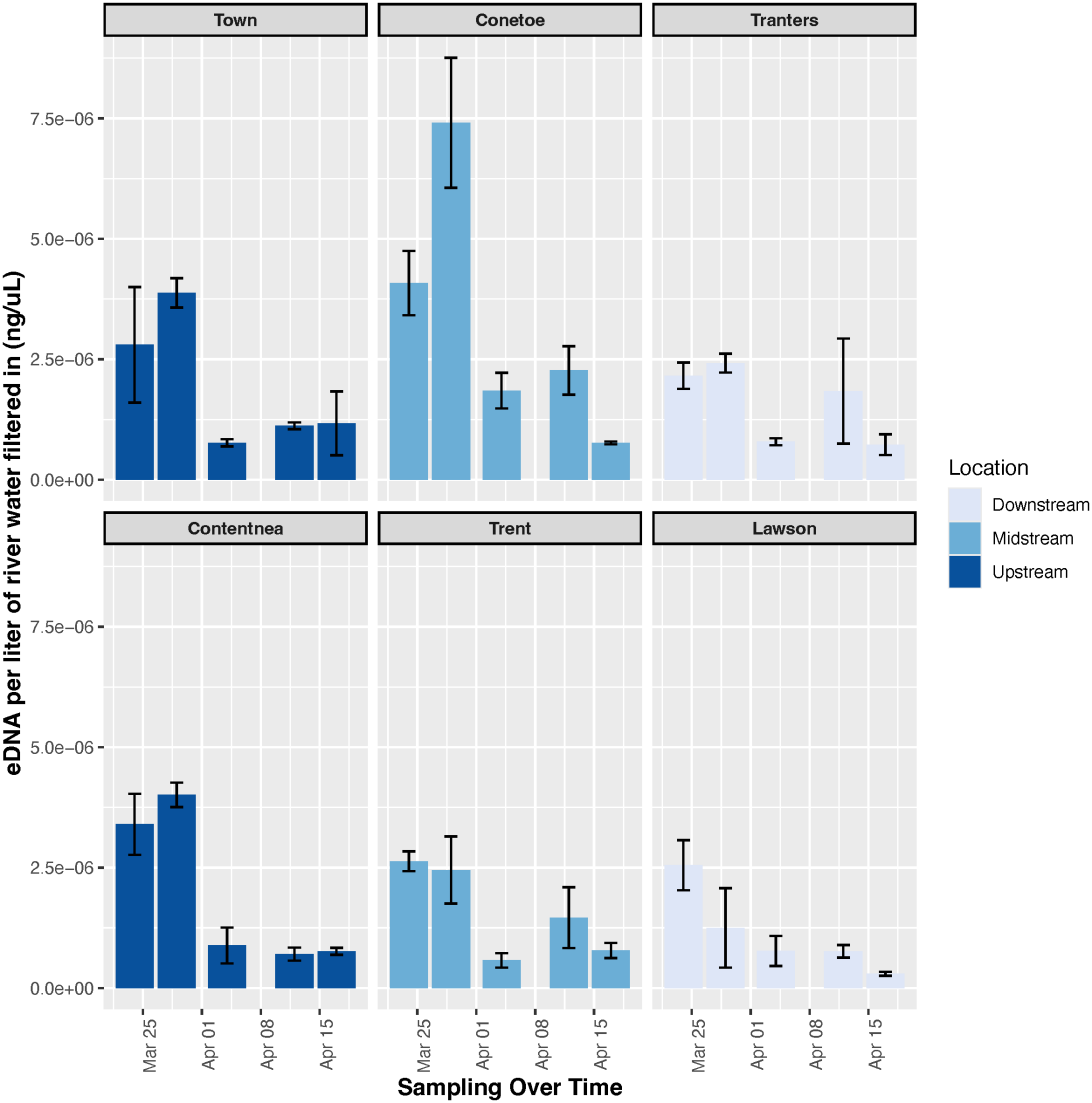
eDNA concentrations across the Tar-Pamlico River (top) and the Neuse River (bottom) 2019 spawning season in relation to their distance from the mouth of the river (upstream left to downstream right) indicate that eDNA trends are more consistent between watersheds further inland than towards the mouth of the river. eDNA concentrations have been normalized to ng river herring eDNA per 1L water filtered and standard errors for replicate biological samples are included.

In all three of the Tar-Pamlico River sampling sites the peak eDNA concentration was measured for March 29^th^ (Fig 5, S2 Table, S3 Fig). Conversely, Neuse River sites peaked in eDNA concentrations in the first two weeks of the sampling season, indicating that the spawning run was fully engaged when sampling was initiated. It is also notable that a late-season peak was not clearly visible in the extended sampling efforts as it was in the eDNA concentration and fish count comparison study in the Neuse River. This absence could be further evidence of variability in spawning runs across years and locations, or it is possible that a late-season peak could have occurred after the sampling efforts. In the two further upstream sites in both systems (Town Creek and Conetoe Creek in the Tar-Pamlico, Contentnea Creek and Trent River in the Neuse River) eDNA concentrations were highest during the first two weeks of sampling in all but one of the sites (Trent River). In addition, increases in eDNA concentration were observed for these sites between the first and second sampling week of sampling leading to the peak eDNA concentration in week 2 of our sampling effort overall. The following three sampling dates at these sites were much lower, and all but one site (Contentnea Creek) saw increases in concentration between the third and fourth sampling dates (4/8/2018 and 4/12/2018). These trends are very similar visually, which contrasts with the downstream sites on each river (Tranter’s Creek on the Tar-Pamlico River, Lawson Creek on the Neuse River) which have patterns that appear to be much less similar to one another. The two sites do share decreases in eDNA concentrations between sampling weeks 2 and 3 as well as weeks 4 and 5, but these decreases are in varying amounts and do not appear to point to any specific pattern.

**Fig 6:**
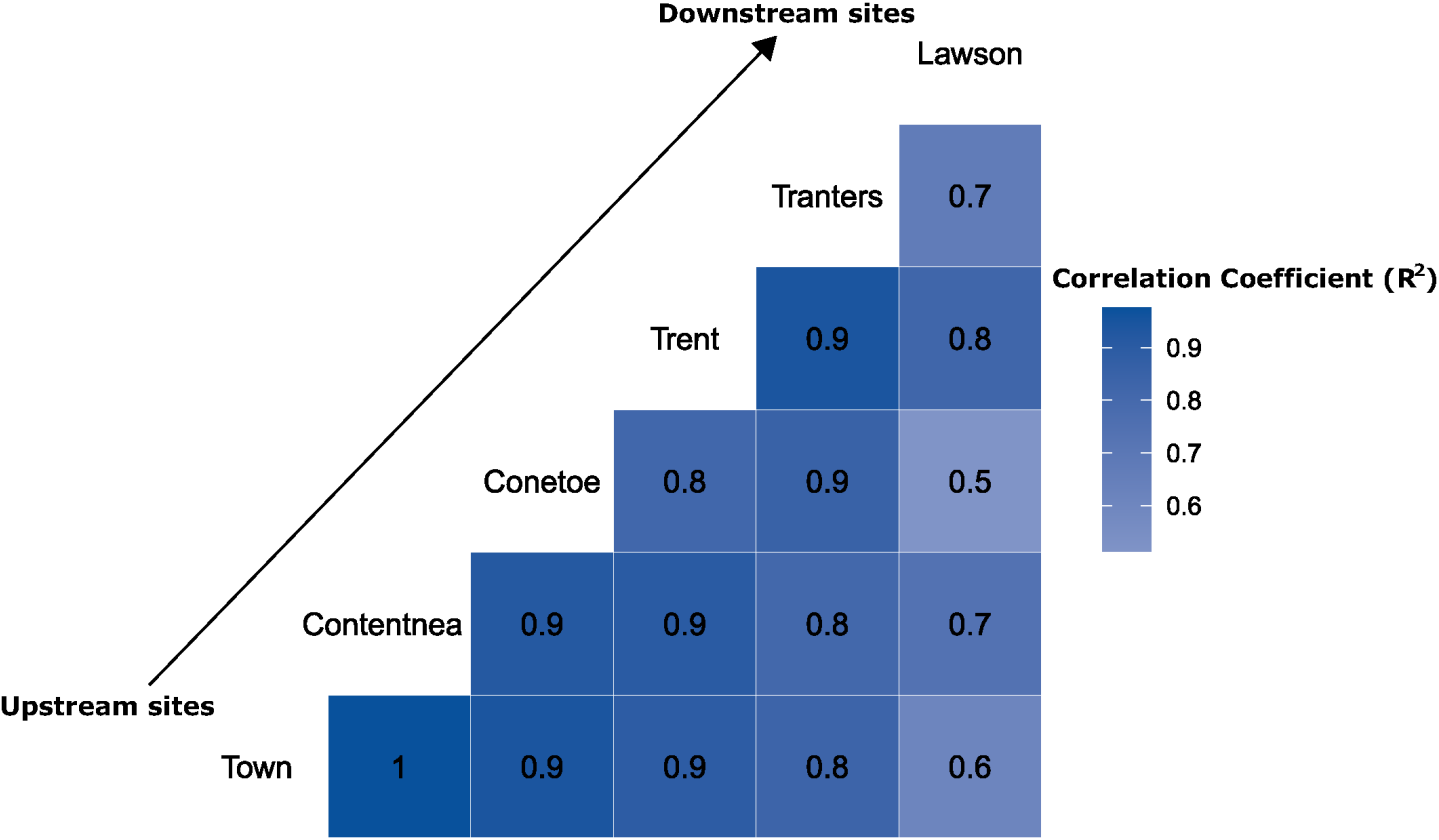
Pearsons’s pairwise correlation analysis indicates statistically significant (p < 0.05) differences between eDNA concentration of multiple sampling locations along five tributaries of the Neuse River (Town Creek, Contentnea Creek, Conetoe Creek, Trent River, Lawson Creek), and one tributary of the Tar-Pamlico (Tranters Creek) over a five-week period in 2019. All pairwise comparisons listed here are significantly correlated (see S3 Table for numerical values; sampling size n = 30).

Correlation analysis results further confirmed that sites that are located at similar distances from the mouths of their respective rivers showed similar eDNA trends, regardless of which of the two river systems they were located on. This finding suggests that a site’s distance from the mouth of the river, which could influence a variety of factors including flow and the amount of influence from estuarine conditions, could be a strong factor affecting eDNA patterns. Lawson Creek, a tributary of the Neuse River, exhibited the least number of similarities to all the other sites that were used in the analysis (Fig 6, S3 Table). Town Creek showed the strongest relationship with Contentnea Creek, the site closest to it in river kilometers and furthest upstream from the sound, followed by Conetoe Creek, Trent River, and Tranters Creek. The Conetoe Creek and Contentnea Creek correlation, which are closer in river kilometers, was greater than the Town Creek Contentnea Creek correlation.

Linear Mixed Effects modeling did not indicate a significant effect of distance upstream. This held true in both categorical (upstream, midstream, and downstream; p = 0.29) measures and absolute distance upstream in km (p = 0.15). Model selection using AIC found that sampling date should not be included in the final model (S3 Fig). Randomization tests of distance values for both categorical and numerical models yielded similar results with p-values of 0.71 and 0.16 respectively.

The weekly sampling on the Tar-Pamlico and Neuse Rivers suggests that, even though there may be variability in the timing of spawning runs, there was some consistency in eDNA trends further upstream. In both the Neuse and Tar-Pamlico Rivers, high eDNA concentrations were measured during the first two weeks of sampling, followed by much lower concentrations for the remainder of the sampling season in the two upstream sites (Town Creek and Conetoe Creek in the Tar-Pamlico, Contentnea Creek and Trent River in the Neuse River) point to patterns present in inland sites within individual spawning seasons. This decline in eDNA concentrations later in the season seems to be reflective of the nature of river herring spawning runs, where many adult river herring enter the system at the very beginning of their spawning season. As the season progresses it is also possible that eDNA degradation is occurring quicker due to increasing temperatures, as both warmer water and the increase in microbial activity it can cause could speed up this process and reduce eDNA detectability. The Town Creek and Conetoe Creek sites resemble each other, as well as the Contentnea Creek and Trent River sites; however, these sites do not bear much resemblance to the sites closer to the mouths of these systems at the Tranter’s Creek and Lawson Creek sites. The two upstream sites had the most similar trends in eDNA, while the middle tributaries showed slightly more variability and the downstream sites were much less consistent overall. It appears that in the sampled tributaries upstream, there were similar trends of eDNA increases and decreases across the spawning season, but there were not consistent trends in sampled tributaries downstream closer to the mouth of the river. One of the reasons for this difference could be that downstream sites could be receiving additional eDNA transported from upstream. Future studies could seek to better understand how far eDNA can travel from its origin down a river system. This difference in the downstream sites could also be due to differences in water flows and influxes due to wind tides from Pamlico Sound that occur since these areas are closer to estuary areas, which have been shown to have an effect on eDNA, or the fact that these rivers are very wide at the mouth and these migrating fish may not be passing by the sampling sites; also, differences in river structure may affect the water flow [40]. More turbulent flow downstream could also be causing inconsistency by dispersing eDNA from the area in which it is shed by the fish. eDNA could possibly be removing eDNA from the water column in these areas through sedimentation. Overall, these results suggest that sampling location within the watershed is important for consistent results and is an important consideration when designing eDNA monitoring plans.

## Conclusions

The results of this study show that eDNA sampling results are more similar to electrofishing results early in the river herring spawning season, but not later on in the season. With this information in mind, it is important to plan additional studies on how larval and juvenile river herring effect measured eDNA in the environment to see how these different life stages have affected this study and how it could affect future studies. It will be important to consider how water flow and environmental factors can change the results when attempting to incorporate eDNA methodologies into monitoring operations in Eastern NC river systems, as well as other river systems nationwide. Studies also suggest that high flow can decrease detectability of environmental DNA, so different events that cause higher than normal levels of flow could affect sampling [41]. Based on the results here that river herring abundances do generally correlate with higher eDNA concentrations it suggests that turbid streams can be monitored using eDNA methodology in the future. The option to use eDNA techniques either in tandem with traditional sampling methods or by itself for river herring population monitoring is one step closer due to these results as we are now aware of some of the basic behaviors of river herring eDNA in the environment.

## Supporting information

S1 Table Neuse River sampling data for Village Creek and Core Creek collected in 2018 and 2019 for both river herring eDNA concentrations and river herring counts collected via electrofishing. (PDF)

S2 Table Tar-Pamlico River and Neuse River sampling data and river herring eDNA concentrations for five tributaries collected across the spring spawning season in 2019. (PDF)

S3 Table Pearsons’s correlation analysis indicates statistically significant differences between eDNA concentration and five tributaries of the Neuse from upstream to downstream (Town Creek, Contentnea Creek, Conetoe Creek, Trent River, Lawson Creek), and one tributary of the Tar (Tranters Creek) over five weeks in 2019. (PDF)

S1 Fig Water samples from (panel A) the Chowan River watershed in the Blackwater River (S1 and S2), a headwater tributary of the Chowan River and at three locations along the mainstem Chowan River (WR, CC, LC) were collected to demonstrate efficacy of the eDNA methodology in NC river systems. River herring presence (panel B) using eDNA was positive in the Blackwater River (S1, S2) and Wicaccon River (WR) and was confirmed by electrofishing (indicated by stars). RH were also present in Catherine’s Creek replicate water samples (CCA, CCB) during April 2017, but not in June 2016 (CC1, CC2) post-spawning season. The lower Chowan River (LC) potentially had some detectable RH eDNA at time of water collection, but this was not confirmed with electrofishing, the band is present in only a single replicate, and the recovered band is weak. Positive controls AL1 and AL2 are fin clips from two Alewife collected April 2017 from Catherine’s Creek.
(PDF)

S2 Fig Individual spawning adult river herring fish abundances and eDNA concentrations across the 2018 and 2019 spawning seasons at the Core Creek (orange lines with markers) and Village Creek (green lines with markers) sites along the Neuse River normalized to calendar day of the year. eDNA concentrations have been normalized to ng river herring eDNA per 1L water filtered and standard errors for replicate biological samples are included.
(PDF)

S3 Fig Linear Mixed Effects modeling did not indicate a significant effect of distance upstream. This held true in both categorical (upstream, midstream, and downstream; p = 0.29) measures and absolute distance upstream in km (p = 0.15). Randomization tests of distance values for both categorical and numerical models yielded similar results with p-values of 0.71 and 0.16 respectively.
(PDF)
