# Supplementary material for: Trends in river herring environmental DNA in two North Carolina river systems
Source: PLoS One. 2026 May 4;21(5):e0347206. doi: 10.1371/journal.pone.0347206 (PMC13138675; doi:10.1371/journal.pone.0347206)
Supplement: S1 Table — (PDF) [file pone.0347206.s001.pdf]

Table S1. Neuse River sampling data for Village Creek and Core Creek collected in 2018 and 2019 for both river herring eDNA concentrations and river herring counts collected via electrofishing.

Sample Date	Sample Site	Average eDNA (ng/uL) per L filtered water	Std Error eDNA (ng/uL) per L filtered water	River Herring Fish Counts
2/26/2018	Village Creek	4.26E-05	1.31E-06	97
3/6/2018	Village Creek	4.37E-05	5.41E-06	34
3/13/2018	Village Creek	1.91E-05	1.73E-06	53
3/20/2018	Village Creek	3.03E-05	3.45E-07	55
3/27/2018	Village Creek	4.34E-05	5.89E-06	25
4/4/2018	Village Creek	4.53E-05	4.47E-06	6
4/11/2018	Village Creek	4.25E-05	3.81E-06	2
2/26/2018	Core Creek	2.46E-05	2.03E-07	0
3/6/2018	Core Creek	1.04E-05	1.34E-06	7
3/13/2018	Core Creek	9.17E-06	4.36E-07	0
3/20/2018	Core Creek	1.64E-05	4.22E-06	27
3/27/2018	Core Creek	1.78E-05	4.84E-07	33
4/4/2018	Core Creek	2.21E-05	5.16E-06	10
4/11/2018	Core Creek	4.41E-05	7.14E-06	2
3/19/2019	Village Creek	2.27E-06	3.88E-08	29
3/28/2019	Village Creek	1.23E-06	2.25E-07	59
4/3/2019	Village Creek	1.33E-06	8.88E-09	22
4/11/2019	Village Creek	9.65E-06	5.03E-07	2
3/19/2019	Core Creek	2.53E-06	3.51E-07	25
3/28/2019	Core Creek	2.91E-07	1.34E-07	30
3/4/2019	Core Creek	8.30E-07	1.20E-08	6
4/11/2019	Core Creek	6.75E-07	8.78E-08	0
