# Supplementary material for: Trends in river herring environmental DNA in two North Carolina river systems
Source: PLoS One. 2026 May 4;21(5):e0347206. doi: 10.1371/journal.pone.0347206 (PMC13138675; doi:10.1371/journal.pone.0347206)
Supplement: S2 Table — (PDF) [file pone.0347206.s002.pdf]

Supplemental Table S2. Tar-Pamlico River and Neuse River sampling data and river herring eDNA concentrations for five tributaries collected across the spring spawning season in 2019.

**Sample Date**	**River System**	**Sample Site**	**Defined Location**	**Average eDNA (ng/uL) per L filtered water**	**Std Error eDNA (ng/uL) per L filtered water**
3/25/2019	Tar-Pamlico	Town Creek	Upstream	2.80E-06	1.20E-06
3/29/2019	Tar-Pamlico	Town Creek	Upstream	3.88E-06	3.05E-07
4/4/2019	Tar-Pamlico	Town Creek	Upstream	7.65E-07	7.41E-08
4/12/2019	Tar-Pamlico	Town Creek	Upstream	1.12E-06	6.98E-08
4/17/2019	Tar-Pamlico	Town Creek	Upstream	1.17E-06	6.64E-07
3/24/2019	Tar-Pamlico	Conetoe Creek	Midstream	4.08E-06	6.67E-07
3/29/2019	Tar-Pamlico	Conetoe Creek	Midstream	7.41E-06	1.35E-06
4/4/2019	Tar-Pamlico	Conetoe Creek	Midstream	1.85E-06	3.71E-07
4/12/2019	Tar-Pamlico	Conetoe Creek	Midstream	2.27E-06	5.03E-07
4/17/2019	Tar-Pamlico	Conetoe Creek	Midstream	7.66E-07	2.91E-08
3/24/2019	Tar-Pamlico	Tranters Creek	Downstream	2.16E-06	2.74E-07
3/29/2019	Tar-Pamlico	Tranters Creek	Downstream	2.42E-06	1.95E-07
4/4/2019	Tar-Pamlico	Tranters Creek	Downstream	7.90E-07	7.27E-08
4/12/2019	Tar-Pamlico	Tranters Creek	Downstream	1.84E-06	1.09E-06
4/17/2019	Tar-Pamlico	Tranters Creek	Downstream	7.26E-07	2.15E-07
3/24/2019	Neuse	Contentnea Creek	Upstream	3.40E-06	6.33E-07
3/29/2019	Neuse	Contentnea Creek	Upstream	4.01E-06	2.52E-07
4/4/2019	Neuse	Contentnea Creek	Upstream	8.85E-07	3.73E-07
4/12/2019	Neuse	Contentnea Creek	Upstream	7.06E-07	1.35E-07
4/17/2019	Neuse	Contentnea Creek	Upstream	7.62E-07	7.41E-08
3/24/2019	Neuse	Trent Creek	Midstream	2.63E-06	2.05E-07
3/29/2019	Neuse	Trent Creek	Midstream	2.45E-06	6.95E-07
4/4/2019	Neuse	Trent Creek	Midstream	5.74E-07	1.48E-07
4/12/2019	Neuse	Trent Creek	Midstream	1.46E-06	6.32E-07
4/17/2019	Neuse	Trent Creek	Midstream	7.80E-07	1.56E-07
3/24/2019	Neuse	Lawson Creek	Downstream	2.55E-06	5.21E-07
3/29/2019	Neuse	Lawson Creek	Downstream	1.25E-06	8.24E-07
4/4/2019	Neuse	Lawson Creek	Downstream	7.69E-07	3.10E-07
4/12/2019	Neuse	Lawson Creek	Downstream	7.62E-07	1.31E-07
4/17/2019	Neuse	Lawson Creek	Downstream	2.95E-07	3.98E-08
