# Supplementary material for: Trends in river herring environmental DNA in two North Carolina river systems
Source: PLoS One. 2026 May 4;21(5):e0347206. doi: 10.1371/journal.pone.0347206 (PMC13138675; doi:10.1371/journal.pone.0347206)
Supplement: S3 Table — (PDF) [file pone.0347206.s003.pdf]

Supplemental Table S3. Pearsons's correlation analysis indicates statistically significant differences between eDNA concentration and five tributaries of the Neuse from upstream to downstream (Town Creek, Contentnea Creek, Conetoe Creek, Trent River, Lawson Creek), and one tributary of the Tar (Tranters Creek) over five weeks in 2019.

	**Town Creek**	**Contentnea Creek**	**Conetoe Creek**	**Trent River**	**Tranters Creek**	**Lawson Creek**
**Town Creek**	1					
**Contentnea Creek**	0.9755***	1				
**Conetoe Creek**	0.9448***	0.9181***	1			
**Trent River**	0.8923***	0.9042***	0.8183***	1		
**Tranters Creek**	0.8344***	0.8103***	0.8518***	0.9450***	1	
**Lawson Creek**	0.6091***	0.7410***	0.5134***	0.8234***	0.6724***	1

*** indicate p values that are $p<0.001$.
