# Supplementary material for: Trends in river herring environmental DNA in two North Carolina river systems
Source: PLoS One. 2026 May 4;21(5):e0347206. doi: 10.1371/journal.pone.0347206 (PMC13138675; doi:10.1371/journal.pone.0347206)
Supplement: S1 Fig — River herring presence (panel B) using eDNA was positive in the Blackwater River (S1, S2) and Wicaccon River (WR) and was confirmed by electrofishing (indicated by stars). RH were also present in Catherine’s Creek replicate water samples (CCA, CCB) during April 2017, but not in June 2016 (CC1, CC2) post-spawning season. The lower Chowan River (LC) potentially had some detectable RH eDNA at time of water collection, but this was not confirmed with electrofishing, the band is present in only a single replicate, and the recovered band is weak. Positive controls AL1 and AL2 are fin clips from two Alewife collected April 2017 from Catherine’s Creek. (PDF) [file pone.0347206.s004.pdf]

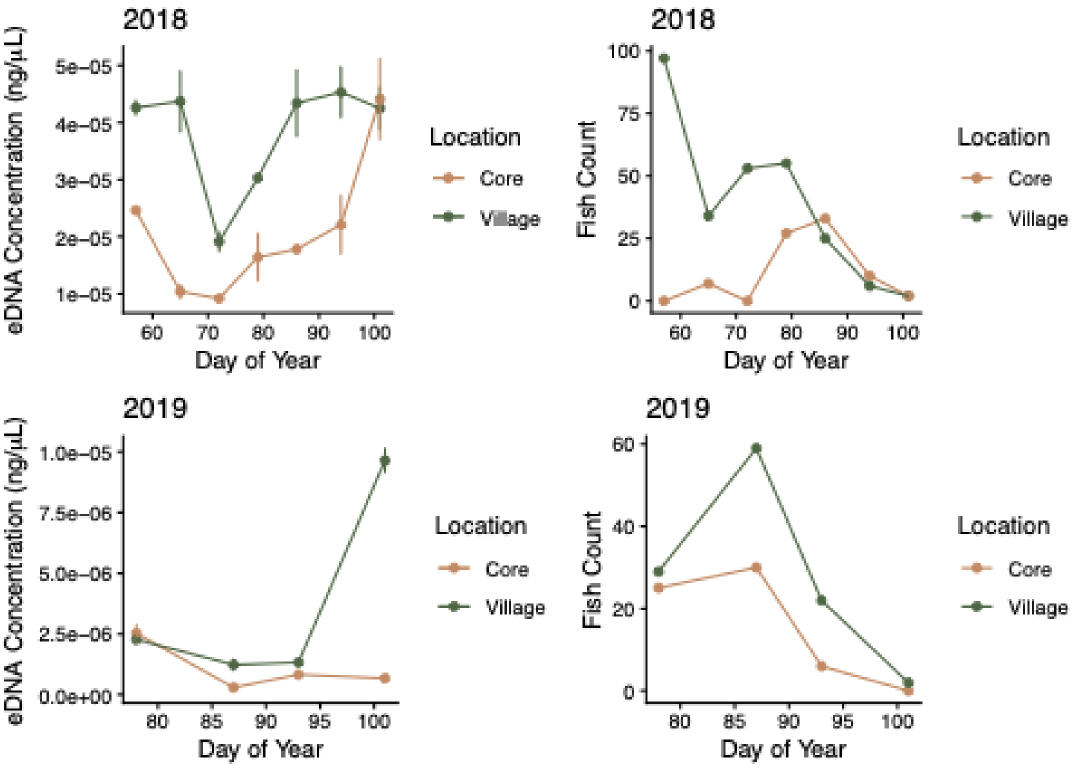

Figure S1. Individual spawning adult river herring fish abundances and eDNA concentrations across the 2018 and 2019 spawning seasons at the Core Creek (orange lines with markers) and Village Creek (green lines with markers) sites along the Neuse River normalized to calendar day of the year. eDNA concentrations have been normalized to ng river herring eDNA per 1L water filtered and standard errors for replicate biological samples are included.
