# Supplementary material for: Trends in river herring environmental DNA in two North Carolina river systems
Source: PLoS One. 2026 May 4;21(5):e0347206. doi: 10.1371/journal.pone.0347206 (PMC13138675; doi:10.1371/journal.pone.0347206)
Supplement: S2 Fig — eDNA concentrations have been normalized to ng river herring eDNA per 1L water filtered and standard errors for replicate biological samples are included. (PDF) [file pone.0347206.s005.pdf]

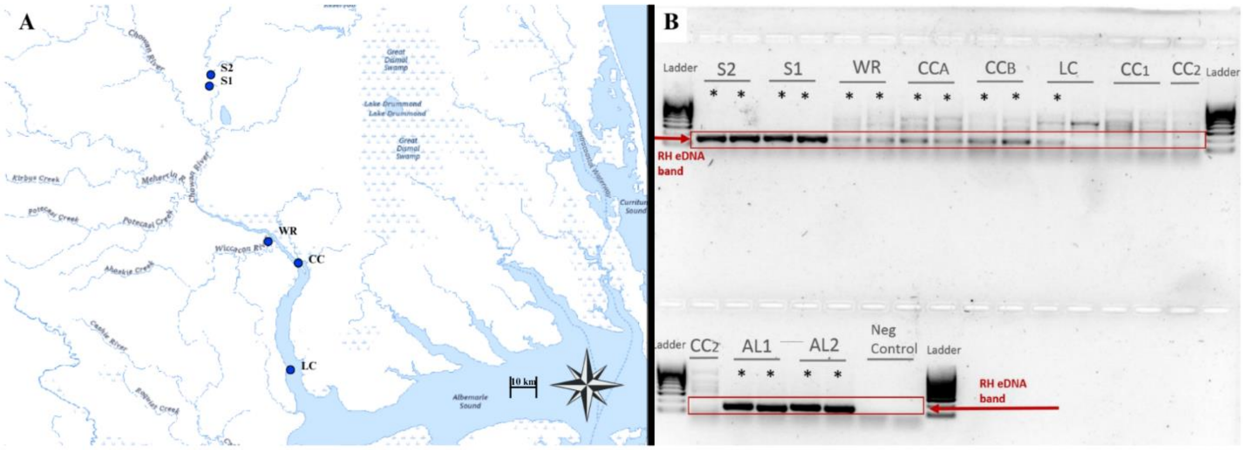

Figure S2. Water samples from (panel A) the Chowan River watershed in the Blackwater River (S1 and S2), a headwater tributary of the Chowan River and at three locations along the mainstem Chowan River (WR, CC, LC) were collected to demonstrate efficacy of the eDNA methodology in NC river systems. River herring presence (panel B) using eDNA was positive in the Blackwater River (S1, S2) and Wicaccon River (WR), and was confirmed by electrofishing (indicated by stars). RH were also present in Catherine's Creek replicate water samples (CCA, CCB) during April 2017, but not in June 2016 (CC1, CC2) post-spawning season. The lower Chowan River (LC) potentially had some detectable RH eDNA at time of water collection, but this was not confirmed with electrofishing, the band is present in only a single replicate, and the recovered band is weak. Positive controls AL1 and AL2 are fin clips from two Alewife collected April 2017 from Catherine's Creek. Image from the U.S. Geological Survey's Geospatial Program, The National Map [41] modified with MS Paint tool (Windows 11).
