# Supplementary material for: Trends in river herring environmental DNA in two North Carolina river systems
Source: PLoS One. 2026 May 4;21(5):e0347206. doi: 10.1371/journal.pone.0347206 (PMC13138675; doi:10.1371/journal.pone.0347206)
Supplement: S3 Fig — This held true in both categorical (upstream, midstream, and downstream; p = 0.29) measures and absolute distance upstream in km (p = 0.15). Randomization tests of distance values for both categorical and numerical models yielded similar results with p-values of 0.71 and 0.16 respectively. (PDF) [file pone.0347206.s006.pdf]

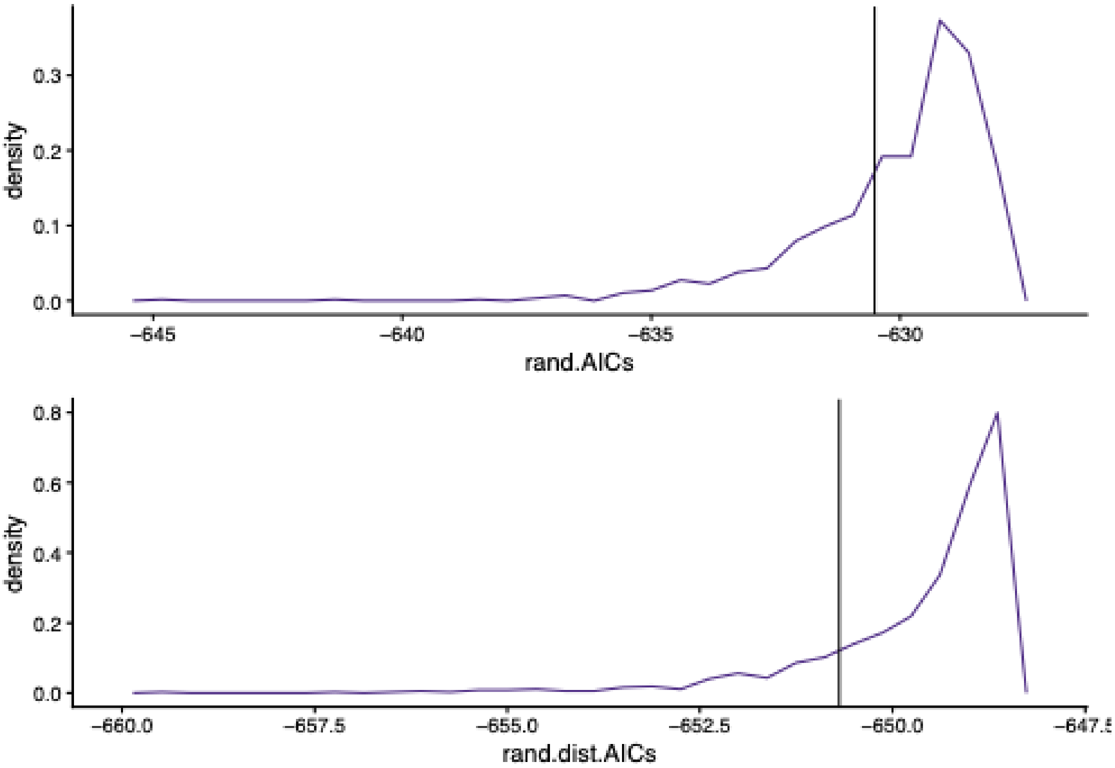

Figure S3. Linear Mixed Effects modeling did not indicate a significant effect of distance upstream. This held true in both categorical (upstream, midstream, and downstream; p = 0.29) measures and absolute distance upstream in km (p = 0.15). Randomization tests of distance values for both categorical and numerical models yielded similar results with p-values of 0.71 and 0.16 respectively.
